# Adverse events after nivolumab and ipilimumab combined immunotherapy in advanced renal cell carcinoma: a multicentre experience in Poland

**DOI:** 10.1186/s12885-024-13192-8

**Published:** 2024-11-15

**Authors:** Renata Pacholczak-Madej, Artur Drobniak, Łukasz Stokłosa, Anna Bidas, Jolanta Dobrzańska, Aleksandra Grela-Wojewoda, Agnieszka Roman, Daria Tusień-Małecka, Jerzy Walocha, Paweł Blecharz, Mirosława Puskulluoglu

**Affiliations:** 1 Department of Chemotherapy, The District Hospital, Sucha Beskidzka, Poland; 2 Department of Gynecological Oncology, Krakow Branch, Maria Sklodowska-Curie National Research Institute of Oncology, Garncarska 11 Street, Krakow, 31-115 Poland; 3 Department of Anatomy, Medical College, Jagiellonian University, Krakow, Poland; 4 Department of Chemotherapy, The Specialistic Hospital, Nowy Targ, Poland; 5 Department of Clinical Oncology, Holy Cross Cancer Center, Kielce, Poland; 6 Department of Oncology, Jagiellonian University Medical College, Krakow, Poland; 7 Oncology Clinical Department, The University Hospital, Krakow, Poland; 8 Department of Clinical Oncology, Maria Sklodowska-Curie National Research Institute of Oncology, Krakow Branch, Krakow, Poland; 9 Department of Clinical Oncology, Ludwik Rydygier Hospital, Krakow, Poland; 10 Clinical and Experimental Oncology Clinic, Institute of Oncology, Karol Marcinkowski Medical University, Poznań, Poland

**Keywords:** Renal cell carcinoma, Immunotherapy, Nivolumab and ipilimumab, Adverse events, Survival analysis

## Abstract

**Background:**

Immune checkpoint inhibitors (ICIs) have been employed in the adjuvant and metastatic setting of renal cell carcinoma (RCC) treatment. Among ICIs, combined immunotherapy has the highest risk for immune-related adverse events (irAEs). We aimed to document the incidence of irAEs in RCC patients treated with nivolumab and ipilimumab as data from the European population remain limited.

**Materials and methods:**

We analysed data from 88 RCC patients treated with nivolumab + ipilimumab between May 2022 and June 2024 across six high-volume oncology units in Poland. We reviewed irAEs and estimated their impact on survival parameters via univariate and multivariate Cox proportional hazards regression models, along with log-rank tests.

**Results:**

With a median follow-up of 11.3 months, the median overall survival (OS) was not reached, whereas the median progression-free survival (PFS) was 12.8 months (6.3–19.3). A total of 74 irAEs were recorded in 50 patients. The most frequent events were endocrine (*n* = 20, 27%), hepatic (*n* = 15, 17%), general (*n* = 12, 13.6%), and cutaneous (*n* = 11, 12.5%). The occurrence of irAEs was associated with a 60% lower risk of disease progression (hazard ratio 0.44, 95% confidence interval 0.2–0.87, *p* = 0.018) without impacting OS and higher disease control rate (*n* = 45, 90% vs. *n* = 24, 63.2%, *p* = 0.004). In contrast, patients with hepatotoxicity had poorer outcomes, with a 2.6-fold greater risk of death (*p* = 0.05).

**Conclusions:**

IrAEs may serve as a predictive factor for the efficacy of the nivolumab + ipilimumab regimen in RCC patients. Special attention is needed for hepatotoxicity, as it can significantly impact survival outcomes.

**Supplementary Information:**

The online version contains supplementary material available at 10.1186/s12885-024-13192-8.

## Introduction

Immunotherapy involving immune checkpoint inhibitors (ICIs), such as anti-programmed cell death receptor/ligand 1 (PD-1/PD-L1) and anti-cytotoxic T lymphocyte-associated antigen 4 (CTLA-4) antibodies, plays a well-established role in the treatment of clear cell renal cell carcinoma (RCC). Initially, nivolumab, an anti-PD-1 antibody, was recommended as a second-line treatment for advanced/metastatic RCC following progression on antiangiogenic therapy [1]. Subsequently, immunotherapy has been introduced as a first-line treatment, either as a combination of nivolumab and ipilimumab (an anti-CTLA-4 antibody) for patients at intermediate and poor risk according to the International Metastatic RCC Database Consortium (IMDC) prognostic index or with anti-PD-1/anti-PD-L1 antibodies in combination with antiangiogenic therapy across all IMDC patient groups [2]. More recently, pembrolizumab, another anti-PD-1 antibody, has been approved for adjuvant treatment in high-risk patients (as defined in the trial protocol), demonstrating benefits in overall survival (OS) and disease-free survival [3].

As a result, immune-related adverse events (irAEs) have become common in the management of RCC in both adjuvant and metastatic settings. Combined immunotherapy is associated with a greater incidence of serious irAEs, occurring in 46% of patients in the pivotal Checkmate 214 trial [4] compared to 19% of patients receiving nivolumab monotherapy in the Checkmate 025 trial [1]. Evidence from other malignancies suggests that the occurrence of irAEs may influence patient prognosis and serve as a prognostic marker for immunotherapy response [5–7]. However, treatment regimens vary among tumors, including dosages and infusion intervals. Additionally, existing real-world studies (RWS) on irAEs in RCC patients treated with immunotherapy have predominantly been conducted in the Japanese population, highlighting the need for further research [8–14].

These disparities underscore the rationale for our RWS. In Poland, the combination of nivolumab and ipilimumab for first-line treatment of metastatic RCC has been reimbursed since May 2022 under specific regulatory criteria, as outlined in the Supplementary Materials. To date, such a study has not been conducted in Polish or broader Caucasian populations. We aimed to document the incidence of irAEs in RCC patients treated with nivolumab and ipilimumab across diverse populations from multiple high-volume oncology units in Poland. Furthermore, we sought to assess the impact of irAEs on treatment outcomes.

## Materials and methods

### Patients and data collection

We conducted a cohort study involving patients with advanced RCC who received first-line combination immunotherapy under a national reimbursement program [15]. The study included patients treated with nivolumab and iplimumab (administered between May 1, 2022, and April 23, 2024), across six oncology units in Poland. The data cut-off was June 30, 2024.

Patients were eligible for inclusion if they met at least one of the following criteria (whichever occurred first): (1) had a computer tomography (CT) scan after treatment initiation, (2) developed toxicity warranting treatment discontinuation, (3) experienced clinical disease progression (PD) with a decline in clinical condition necessitating treatment withdrawal, or (4) died. The reimbursement policy criteria for Poland are detailed in the Supplementary Materials.

The study had an ambispective design. Data on disease diagnosis, initial radical treatment, metastasis sites, and patient demographics and comorbidities (definitions in the Supplementary Materials) were collected retrospectively. Prospective data collection included the timeline of nivolumab + ipilimumab administration, treatment response, and adverse events.

### Study objectives

The primary objective was to evaluate the frequency and severity of irAEs and their impact on survival parameters (OS and progression-free survival [PFS]) in an RWS of RCC patients treated with the nivolumab + ipilimumab regimen. The secondary objective was to assess treatment efficacy by determining the overall response rate (ORR), disease control rate (DCR), and survival parameters (OS, PFS).

Additionally, we aimed to discuss these results with pivotal trials and other RWS. To achieve this goal, we conducted a comprehensive search of the PubMed and Embase databases via the following keywords: renal cell carcinoma OR renal-cell carcinoma OR RCC AND metastatic OR advanced AND immunotherapy OR nivolumab + ipilimumab OR nivolumab and ipilimumab OR combined immunotherapy. We also included conference abstracts reporting unpublished data.

### Treatment regimen

The treatment followed the European Union’s summary of product characteristics [8, 9]: 4 cycles of ipilimumab at 1 mg/kg intravenously (iv) + nivolumab at 3 mg/kg given iv every 3 weeks on the same day. Patients subsequently received maintenance therapy: 240 mg of nivolumab every 2 weeks or 480 mg of nivolumab every 4 weeks until PD, death, the occurrence of unacceptable toxicity, or withdrawal of patient consent. No dose modifications or specific premedication were permitted.

### Evaluation of treatment efficacy

The patient’s responses to treatment were assessed via CT scans of appropriate areas, following the Response Evaluation Criteria in Solid Tumors 1.1 (RECIST 1.1) or immune RECIST (iRECIST) criteria [16], which were conducted every 12 weeks or upon clinical suspicion of PD (as specified by reimbursement criteria [15]; details are provided in the Supplementary Materials). Treatment efficacy was determined by analysing OS and PFS. Additionally, the ORR and DCR were evaluated according to RECIST 1.1 [16]. Definitions of the survival parameters are provided in the Supplementary Materials.

### Evaluation of irAEs

The irAEs that occurred from the start of combined immunotherapy until the end of the observation period were documented and graded according to the Common Terminology Criteria for Adverse Events (CTCAE) version 5.0 [13]. The irAEs were categorized into eight groups: endocrine, hepatic, pulmonary, general (including fatigue, infusion reactions, fever), cutaneous, diarrhea/colitis, rheumatoid, and hematologic.

### Ethical considerations

The study protocol was approved by the Bioethics Committee of Jagiellonian University Medical College (decision number 118.0043.1.115.2024 dated April 19, 2024). Standard institutional informed consent was obtained from each patient before initiating the nivolumab + ipilimumab treatment. All procedures for diagnosing and managing irAEs followed the European Society for Medical Oncology guidelines (ESMO) [14].

### Statistical analysis

Statistical analyses were conducted using PS Imago Pro 9 (SPSS). Categorical variables were compared via Fisher’s exact test or chi-square tests, whereas continuous variables were analysed via the Mann‒Whitney test. PFS and OS were estimated and visualized using the Kaplan‒Meier method. Univariate and multivariate Cox proportional hazards regression models and log-rank tests were used to assess prognostic parameters affecting PFS and OS. Variables with p-values < 0.05 in univariate analyses were included in multivariate analyses. A p-value < 0.05 was considered statistically significant.

## Results

### Patients’ characteristics

The median age of the enrolled patients was 64 years (interquartile range [IQR] 57–73) with 79.5% of them being males (*n* = 70). In total, 61 (69.3%) patients had IMDC intermediate-risk, and 27 (30.7%) had poor risk. The cohort included clear-cell histology with 16% of them presenting sarcomatoid components. Most patients had a performance status (PS) of 1 (75%) at the start of treatment. Half of them had primary metastatic disease (52.3%), with over 80% having undergone nephrectomy. The most common metastatic sites were the lungs, lymph nodes, and bones. The detailed baseline characteristics are presented in Table 1 with additional information regarding comorbidities and sites of metastasis in the Supplementary Materials Table 1S.

**Table 1:** Baseline clinical characteristics of the enrolled patients: comparison of those with and without immune-related adverse events

		All patients *n* = 88	irAEs *n* = 50	no-irAEs *n* = 38	*p*-value
Performance status, n(%)	0	9 (10.2)	4 (8.0)	5 (13.2)	0.36
	1	66 (75.0)	38 (76.0)	28 (73.7)	0.53
	2	13 (14.8)	8 (16.0)	5 (13.2)	0.5
Nephrectomy, n(%)	Yes	71 (80.7)	39 (78.0)	32 (84.2)	0.17
	No	17 (19.3)	11 (22.0)	6 (15.8)	0.37
Time from nephrectomy to treatment initiation (months)		3.4 (1.7–7.7)	3.5 (1.7–10.3)	3.1 (1.7–5.9)	0.5
T stage after nephrectomy according to AJCC 8th edition, n(%)	T1	8 (9.1)	6 (12.0)	2 (5.3)	0.27
	T2	9 (10.2)	5 (10.0)	4 (10.5)	0.6
	T3	47 (53.4)	26 (52.0)	21 (55.3)	0.5
	T4	4 (4.5)	3 (6.0)	1 (2.6)	0.4
	No data	20 (22.7)	10 (20.0)	10 (26.3)	0.38
Histologic grade, n(%)	G1	3 (3.4)	1 (2.0)	2 (5.3)	0.4
	G2	16 (18.2)	7 (14.0)	9 (23.7)	0.24
	G3	19 (21.6)	13 (26.0)	6 (15.8)	0.25
	G4	31 (35.2)	20 (40.0)	11 (28.9)	0.3
	No data	19 (21.6)	9 (18.0)	10 (26.3)	0.3
Primary metastatic, n(%)		46 (52.3)	23 (46.0)	23 (60.5)	0.2
Number of disease sites, n(%)	≤ 2	51 (58.0)	31 (62.0)	20 (52.6)	0.39
	> 2	37 (42.0)	19 (38.0)	18 (47.4)	0.36
IMDC risk group, n(%)	Intermediate	61 (69.3)	37 (74.0)	24 (63.2)	0.38
	Poor	27 (30.7)	13 (26.0)	14 (36.8)	0.28
Number of risk factors, n(%)	1	33 (37.5)	21 (42.0)	12 (31.6)	0.7
	2	28 (31.8)	16 (32.0)	12 (31.6)	0.58
	3	23 (26.1)	11 (22.0)	12 (31.6)	0.3
	4	4 (4.5)	2 (4.0)	2 (5.3)	0.59
No of patients with risk categories, n(%)	Time from the diagnosis to treatment onset < 1 year	76 (86.3)	42 (84.0)	34 (89.5)	0.54
	Karnofsky Score < 80%	19 (21.6)	12 (24.0)	7 (18.4)	0.61
	Hemoglobin level < unl	53 (60.2)	29 (58.0)	24 (63.2)	0.67
	Corrected calcium > unl	7 (8.0)	2 (4.0)	5 (13.2)	0.23
	Neutrophils > unl	5 (5.7)	2 (4.0)	3 (7.9)	0.65
	Platelets > unl	19 (21.6)	10 (20.0)	9 (23.7)	0.8

Categorical variables are presented as numbers (percentages), and continuous variables are presented as medians and interquartile ranges. *Abbreviations* AJCC, American Joint Committee on Cancer; CT, computed tomography; DC, International Metastatic Renal Cell Carcinoma Database Consortium; n, number; T, tumor; unl, upper normal limit

### Safety

**Fig. 1:**
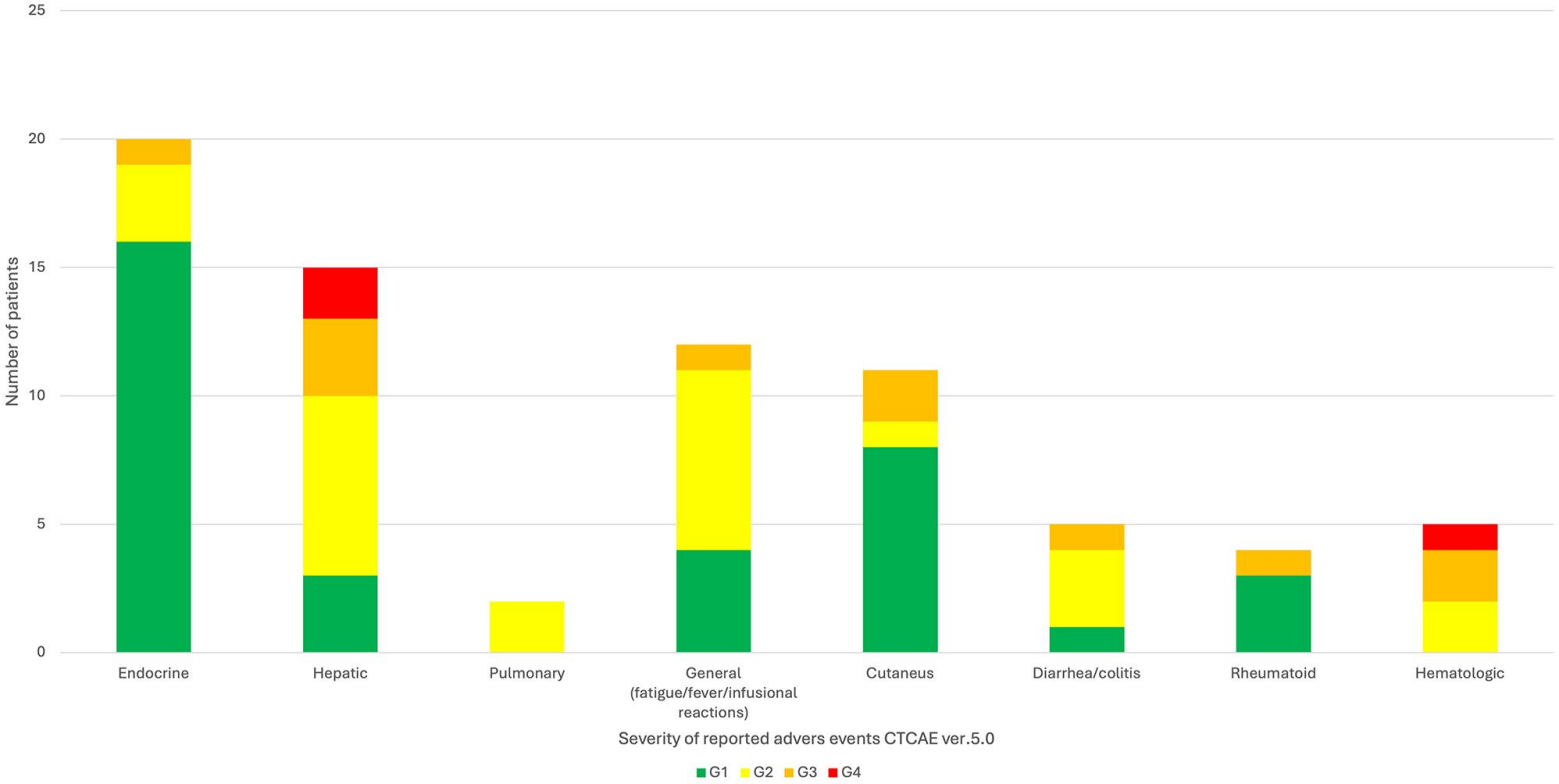
The frequency and distribution of recorded immune-related adverse events. Abbreviations CTCA-Common Terminology Criteria for Adverse Events, G-Grade

In total, 50 patients (56.8%) experienced irAEs, with 74 events recorded (Table 2). Patients with and without irAEs were comparable in baseline characteristics, except for the number of venous thromboembolic events, which differed significantly (*p* = 0.03) (Table 1). Grade (G) 1 irAEs occurred in 35 patients (70% of individuals with irAEs), G2 in 25 (50%), G3 in 11 (22%), and G4 in 3 individuals (6%). Overall, in the whole group, 20 patients (22.7%) experienced multiple irAE episodes (2 episodes in *n* = 12, 13.6%; 3 episodes in *n* = 7, 8%; and 4 episodes in *n* = 1, 1%). The most frequent irAEs were endocrine-related (*n* = 20, 27%), followed by hepatotoxicity (*n* = 15, 17%), general disorders (*n* = 12, 13.6%), and cutaneous events (*n* = 11, 12.5%) (Fig. 1). The median time to onset of irAEs was 2 months (IQR:1.5-3), with 16% of them (*n* = 12) occurring within the first month. Immunosuppressive treatment, primarily steroids at a median dose of 1 mg/kg (IQR: 0.5-1), was administered to 19 patients (38%). Mycophenolate mofetil was used in 2 patients—one for hepatotoxicity and the other for recurrent fever. Twelve patients (13.6%) discontinued treatment because of irAEs, with hepatotoxicity being the most common cause.

**Table 2:** Summary of recorded immune-related adverse events (*n* = 74 events)

		Any grades, (*n*%)	Grade 3–4 *n* = 14	Steroid use *n* = 19	Discontinuation *n* = 12
Endocrine	Hypo/hyperthyroidism, n(%)	16 (18.2)	0	0	0
	Hypophysitis, n(%)	3 (2.3)	1	2	0
	Adrenal insufficiency, n(%)	1 (1.1)	0	0	0
Hepatic, n(%)		15 (17)	5	8	4
Pulmonary, n(%)		2 (2.3)	0	1	0
General	Fatigue, n(%)	8 (9.1)	1	0	1
	Infusional-related reactions, n(%)	2 (2.3)	0	0	0
	Fever, n(%)	2 (2.3)	0	1	1
Cutaneous, n(%)		11 (12.5)	2	2	1
Diarrhea/colitis, n(%)		5 (5.7)	1	2	2
Rheumatoid, n(%)		2 (2.3)	0	1	0
Hematologic	Anemia, n(%)	3 (3.4)	2	1	2
	Neutropenia, n(%)	1 (1.1)	1	0	0
	Thrombocytopenia, n(%)	1 (1.1)	0	0	0
Pericarditis, n(%)		1 (1.1)	0	1	0
Pulmonary embolism, n(%)		1 (1.1)	1	0	1

Abbreviations n-number

### Relationships between irAEs and outcomes

**Fig. 2:**
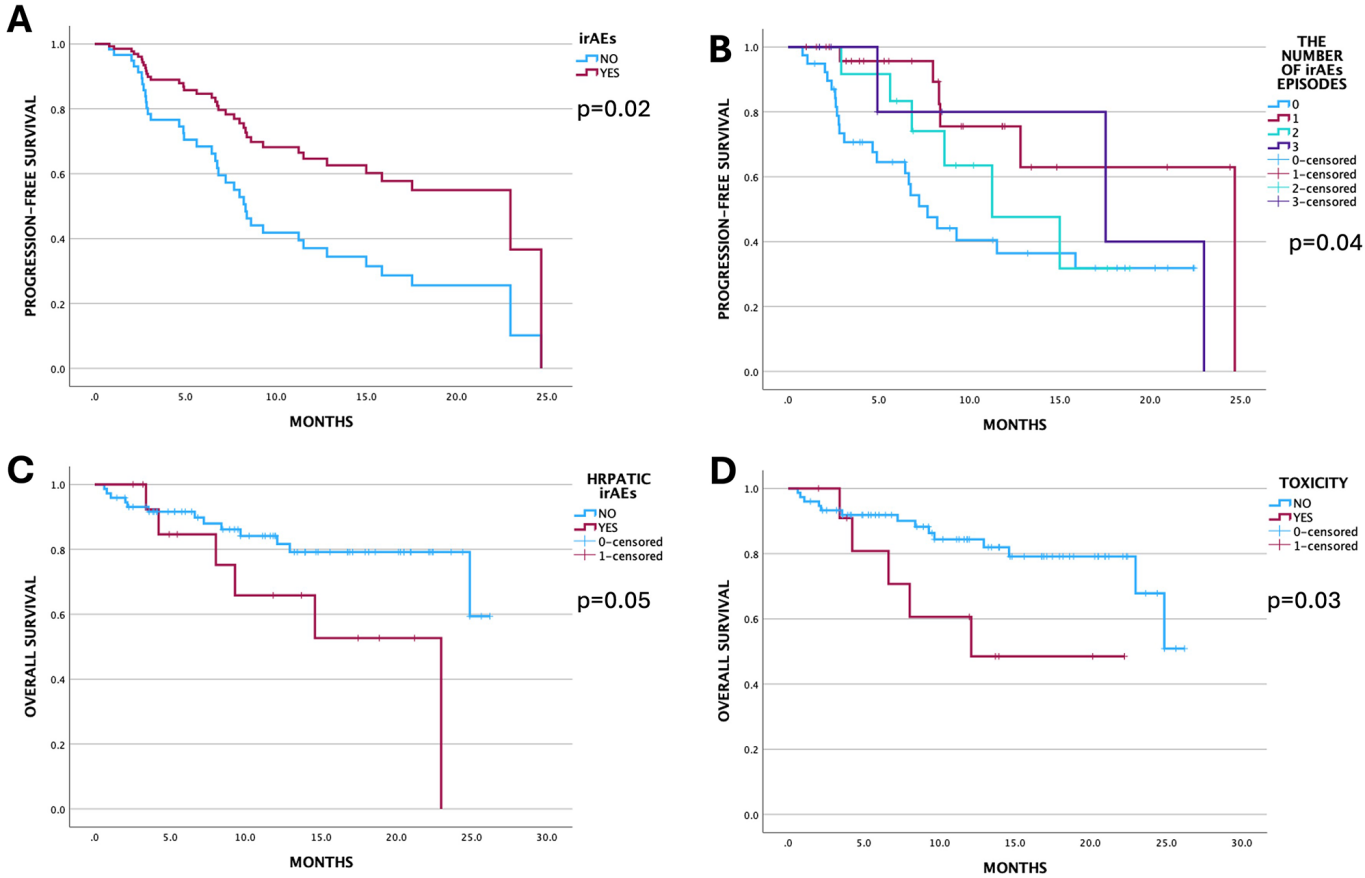
Kaplan-Meier curves of selected parameters influencing treatment outcome: **A**- immune-related adverse events (irAEs) occurrence; **B**- number of recorded irAEs episodes; **C**- hepatic irAEs; **D**- number of patients who discontinued treatment due to irAEs

Among patients who experienced irAEs, the median OS was 24.8 months (21.4–28.3), whereas in those who did not, the median OS was NR (log-rank *p* = 0.87). In contrast, PFS was significantly longer in patients with irAEs (15.5 months [10.2–24.9] vs. 7.2 months [5.3–9.2], *p* = 0.015), with a 60% reduction in the risk of disease progression (HR 0.44, 95% CI 0.2–0.87, *p* = 0.018) (Fig. 2a). The likelihood of longer survival increased with the number of experienced irAEs (log-rank *p* = 0.042, Fig. 2b). Subgroup analysis revealed no difference in OS for patients with endocrine or cutaneous irAEs. However, patients with hepatotoxicity had poorer outcomes (*p* = 0.05), with a 2.6-fold higher risk of death (*p* = 0.05), as indicated in Fig. 2c. Patients who discontinued the treatment due to toxicity had a 3-fold greater risk of death (HR 3, 95% CI 1–8.3, *p* = 0.04) (Fig. 2d).

**Fig. 3:**
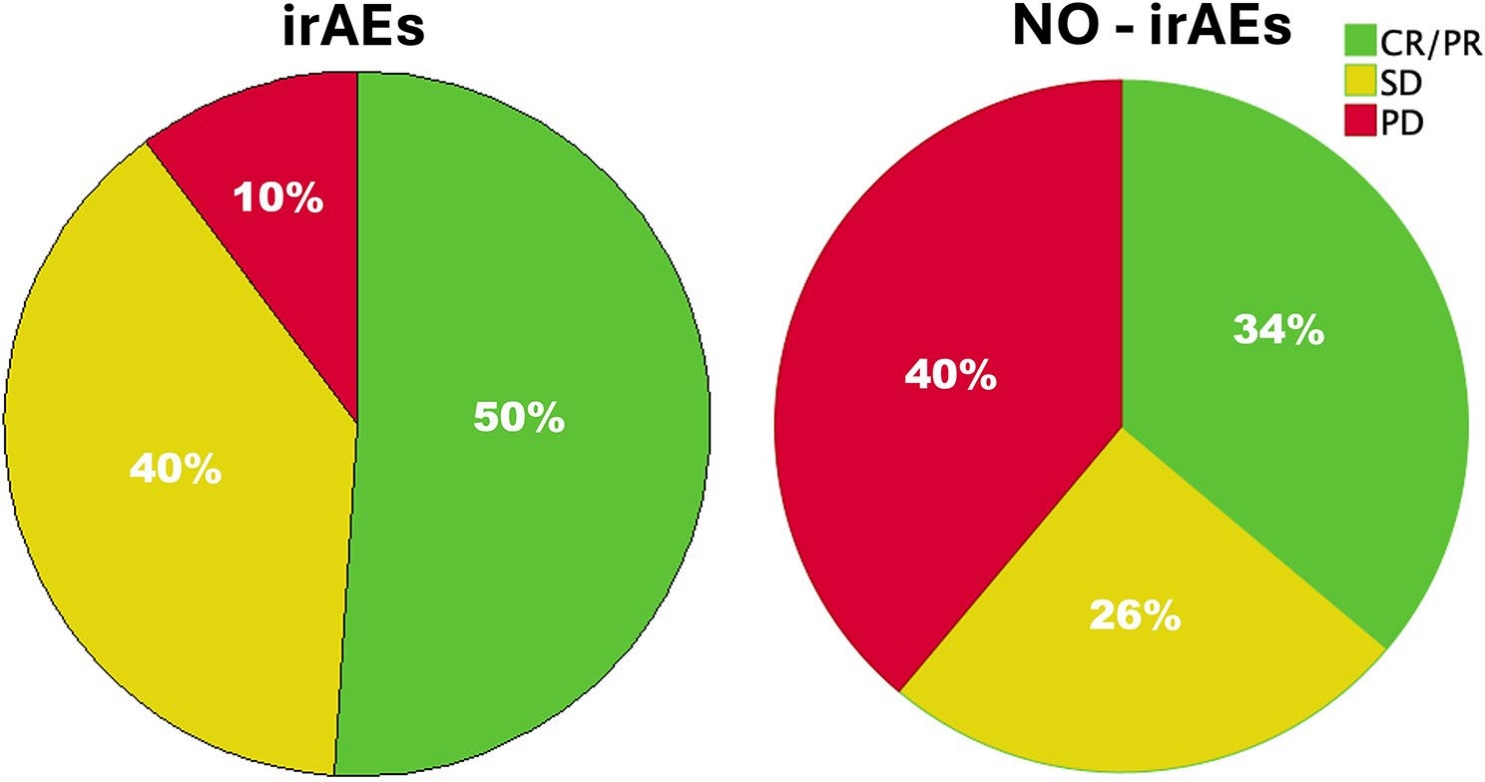
Pie charts of the distribution of radiologic responses in patients with and without immune-related adverse events (irAEs). *Abbreviations* CR/PR- complete remission/partial response, SD- stable disease; PD- progressive disease

Data on the best radiologic responses in patients with irAEs are provided in Fig. 3. The DCR was recorded in 45 (90%) of the patients with toxicities vs. 24 (63.2%) without (*p* = 0.004). On the other hand, the ORR was comparable between these two subgroups (*n* = 25, 50% vs. *n* = 14, 36.8%, *p* = 0.28).

### Clinical outcomes

At the data cut-off after a follow-up period of 11.3 months (IQR: 4.2–17.9), the median duration of immunotherapy was 6.48 months (IQR: 2.7–11.9), with a median of 8 cycles (IQR: 4–14). Eleven patients (12.5%) received fewer than 4 doses due to toxicity or PD. The median OS was not reached (NR) in the overall population, whereas the median PFS was 12.8 months (6.3–19.3). In the IMDC intermediate-risk group, the median OS was 24.8 months (NR-NR), and the PFS was 15.9 months (9.7–22.1). For the IMDC poor-risk group, the median OS was NR, and the PFS was 7.7 months (6.2–9.2). These subgroups were not significantly different (log-rank *p* = 0.053 for OS, *p* = 0.07 for PFS). The number of patients with ORR and DCR in the whole treatment group was 39 (44.3%) and 69 (78.4%), respectively. The ORR in the IMDC intermediate vs. poor risk groups was 27 (44.3%) vs. 12 (44.4%), *p* = 1, whereas the DCR was 52 (85.2%) vs. 17 (63%), *p* = 0.026.

Univariate analysis indicated that the occurrence of irAEs was the only factor affecting PFS (HR 0.4, 95%CI 0.2–0.9, *p* = 0.018), precluding multivariate analysis. However, factors such as PS≥2, hepatotoxicity, and central nervous system (CSN) metastases influence OS. Multivariate analysis (*p* = 0.04 for the model) of these 3 essential predictors revealed that two independent factors, PS≥2 (HR 3.8, 1.4–10.4, *p* = 0.01) and CSN metastases (HR 6.7, 95% CI 1.8–25.5, *p* = 0.005), were significantly associated with OS (Table 2S).

After a median observation period of 11.3 months (IQR: 4.2–17.9), 39 patients (44.3%) continued combined immunotherapy, whereas 49 (55.7%) experienced treatment withdrawal due to PD (*n* = 27, 30.7%), toxicity (*n* = 12, 13.6%), death (*n* = 9, 10.2%) or loss to follow-up (*n* = 1, 1.1%). Among those who had PD (*n* = 39), 24 patients (61.5%) were disqualified from further treatment due to death or poor general condition, 5 (12.8%) were under active observation, and 20 (51.2%) started cabozantinib as second-line therapy.

## Discussion

Our study adds to the literature, confirming that irAEs occurrence is associated with improved outcomes; therefore, toxicity may serve as a predictive marker for the response to immunotherapy. In our analysis, most irAEs occurred within the first 2–3 months of treatment and were typically mild to moderate. Severe irAEs (G3 + G4) were noted in 17% of the enrolled patients, with a 14% discontinuation rate due to these events. Interestingly, steroids were generally sufficient for immunosuppressive treatment. Notably, patients who discontinued treatment owing to irAEs had an increased risk of death, with hepatotoxicity being particularly detrimental, leading to shorter survival and the highest discontinuation rate among irAEs.

Data from the literature concerning irAEs in RCC patients treated with the nivolumab + ipilimumab regimen and their relationships with survival parameters are presented in Table 3. The rate of irAEs reported across all studies varied from 48 to 94%, with the highest incidence observed in the pivotal Checkmate 214 trial [4], likely due to its prospective format and more reliable documentation of such events in clinical trials. In general, our results align with those of other studies, but interestingly, in Checkmate 214, an association between OS and toxicity was not found. In our study, we also did not report the influence of irAEs on OS. However, OS is a multifactorial parameter that can be influenced by various factors, including subsequent lines of therapy and deaths from comorbidities not directly related to cancer progression. Additionally, in real-world clinical practice, factors such as delays in therapy, treatment interruptions, variations in supportive care, and limitations in access to additional therapies may also impact OS in ways that are less predictable than in controlled trial settings [17]. Therefore, PFS is likely a more sensitive indicator of the direct effects of nivolumab + ipilimumab in this context. Similar to the reports of Paderi et al. [18], Hayase et al. [8], and Washino et al. [14], we found that multiple irAEs correlated with better outcomes. However, unlike other findings [8, 11, 18], we did not observe correlations between cutaneous or endocrine events and PFS.

**Table 3:** Summary of the literature on immune-related adverse events in patients with advanced renal-cell carcinoma treated with combined immunotherapy

Type of study	Type of publication	Population	Efficacy	Safety	Influence on outcome	Ref
Randomized, open-lab, phase 3 clinical trial, prospective	Original article	*N* = 547 Age: 62 (6–85)	Follow-up 43.6 mo; mPFS 17 mo (9.7–20.7); mOS 47mo (5.6-NR)	irAEs = 514 pts (94%), 121 events leading to discontinuation (22.1%); fatigue (38%), pruritus (29.3%), diarrhea (28.3%)	OS was similar in irAEs and no-irAEs pts and in pts who discontinued treatment due to toxicity vs. those who did not	[19]
Multicenter, retrospective	Original article	*N* = 324 Age: 62 (24–870 ccRCC = 269 (83%) Italy	Follow-up 12 mo (1–22); mPFS 8.3 (6.5–10.1); mOS NR	irAEs leading to interruption = 55 pts (17%); cumulative irAEs incidence = 274; The most frequent were gastrointestinal, hepatic, pulmonary, toxicities	The use of steroids to treat irAEs did not worsen OS. Pts treated with steroids had better outcome (12-month OS rate of 74.6% vs. 62.5%, *p* = 0.017)	[20]
Multicenter, retrospective	Original article	*N* = 72, Age: 70 (36–86), ccRCC = 55 (76.4%); Japan	Follow-up 16.1 mo (1.4–37.8); mPFS NR (NR); mOS NR (NR)	irAEs = 51 pts (70.8%); 22 pts (30.6%) discontinued due to irAEs; hepatotoxicity (35%), endocrine (29.4%), skin rash (19.6%),	No difference in ORR (*p* = 0.47), PFS (HR 1.1, 95%CI, 0.5–2.5, *p* = 0.36), and OS (HR 0.39, 95%CI, 0.14–1.1, *p* = 0.07) among patients who discontinued treatment due to irAEs	[21]
Multicenter, retrospective	Original article	*N* = 129, Age: 67 (28–87), ccRCC = 107; Japan	Follow-up 12.3 mo; mOS = 28.7 mo; mPFS = 9.9 mo	irAEs = 96 pts (74%); endocrine (28%), skin rash (24%), hepatotoxicity (15%)	Correlation between the development of irAEs and OS (HR 0.32, 95%CI, 1.17–0.65, *p* = 0.001) and PFS (HR 0.33, 95%CI, 0.15–0.74, *p* = 0.007), favourable survival in multiple irAEs, no difference in G1-G2 vs. ≥ G3	[13]
Multicenter, retrospective	Original article	*N* = 69, Age = 65.5 (59–74), ccRCC = 49 (71%); Japan	Follow-up 9.1 mo (2.4–15.5); mOS NR; mPFS 6 mo	irAEs = 38 pts (55%); thyroid dysfunction (23.7%), interstitial pneumonia (21%), skin (18%),	Patients with irAEs vs. no-irAEs had prolonged OS (*p* = 0.012) and PFS (*p* = 0.002)	[12]
Single centre, retrospective	Original article	*N* = 46 Age ≥ 65 (26 pts) ccRCC = 33; Japan	Follow-up 8.5 mo (5.2–15)	irAEs = 33 pts (71.7%); cutaneous (32%), endocrine (33%)	Patients with irAEs vs. no-irAE had prolonged PFS (*p* < 0.0001), but not OS (*p* = 0.57), irAEs were a predictor of longer PFS (HR 0.18, *p* = 0.0005), but not OS; ORRs were higher in pts with irAEs (*p* = 0.0064)	[10]
Single centre, retrospective	Original article	*N* = 43 Age: 64 (45–79) ccRCC = 36 (83.7%) 10 pts (23.7%) received nivolumab + ipilimumab and 33 pts (76.7%) nivolumab monotherapy; Italy	No data	irAEs = 29 pts (67%); endocrine (60%), hepatotoxicity (30%), nephritis (10%)	PFS was longer in patients with thyroid (*p* = 0.028) and cutaneous irAEs (*p* = 0.041). Experiencing ≥2 vs. 0–1 irAEs correlated with a better outcome (HR 0.33, 95%CI, 0.11–0.77, *p* = 0.013)	[18]
Single center, retrospective	Original article	*N* = 35 Age: 66 (42–80) ccRCC = 30 (85.7%); Japan	No data	irAEs = 22 pts (62.9%);	PFS and OS were longer in patients with irAEs (*p* = 0.0012, *p* = 0.015), PFS was longer in pts with cutaneous irAEs (HR 9.3, 95%CI, 1.9–44.5, *p* = 0.005) and adrenal insufficiency (HR 3.6, 95%CI, 1-12.9, *p* = 0.0014)	[11]
Single center, retrospective	Original article	*N* = 91 Age: 67 ccRCC: 75 (82%); Japan; 29 patients received nivolumab + ipilimumab (22%) and 2 nivolumab monotherapy (68%)	Follow-up 27mo (1–49); mOS NR; mPFS 11 mo	irAEs = 44 pts (48%); hepatotoxicity (30%), endocrine (21%), cutaneous (13%) and gastrointestinal (13%)	OS was significantly longer in patients with irAEs (*p* = 0.01). In multivariate analysis, Karnofsky performance status, prior nephrectomy, and irAEs were independent significant predictors of OS.	[22]
Multicenter, retrospective	ESMO 2022 abstract	*N* = 129 Age: 67 (28–87) ccRCC = 107; Japan	Follow-up 12.3 (0.1–36.)	Multisystem irAEs = 39 pts (30%); ≥G2 pituitary (35), thyroid (23), hepatic (14)	Pituitary and thyroid irAEs were associated with improved OS (HR 0.25; *p* = 0.0018; HR 0.35, *p* = 0.039) but no other organ-specific irAEs. Only pituitary irAEs were associated with improved PFS (HR 0.49, *p* = 0.018). Multisystem irAEs were associated with improved survival compared to no or one ≥ Grade 2 irAEs.	[8]

*Abbreviations* ccRCC, clear cell renal cell carcinoma; CI, confidence interval; ESMO, European Society for Medical Oncology; G, grade; HR, hazard ratio; irAEs, immune-related adverse events; mo, months; mOS, median overall survival; mPFS, median progression-free survival; n, number; NR, not reached; ORR, objective response rate; OS, overall survival; O + Y, Opdivo (nivolumab) + Yervoy (ipilimumab); PFS, progression-free survival; pts, patients; vs., versus

The association between irAEs and improved outcomes in RCC patients treated with nivolumab + ipilimumab can be explained by several key biological mechanisms. Abnormal molecular mimicry of antigens shared between tumors and normal tissues can trigger simultaneous T-cell and B-cell cross-reactions, potentially linking latent tissue-specific autoimmunity to both therapy and healthy tissues. Consequently, irAEs may further exacerbate host immune responses, enhance antitumor activity, leading to improved PFS and OS, especially in patients who experience mild to moderate irAEs [23–26]. Pro-inflammatory cytokines, such as interleukin 6 (IL-6) and interferon γ (IFN-γ), are also central to the link between irAEs and outcomes. These cytokines are essential for T-cell activation and proliferation, promoting immune cell recruitment to the tumor microenvironment and strengthening antitumor responses. However, their elevated levels also contribute to systemic inflammation and irAEs. Importantly, higher cytokine levels have been correlated with stronger antitumor responses but also with increased irAE incidence [23, 27]. Self-reactive T-cells, further contribute to irAEs. For example, myocarditis associated with ICIs reflects T-cell infiltration into cardiac tissues, likely due to shared antigen recognition. Additionally, B-cell activation and autoantibody production are linked to irAEs, with autoantibodies frequently observed in patients who develop these events [23, 27, 28]. There are no prospective studies regarding this topic performed for RCC patients population.

In our analysis, 30% of patients who discontinued treatment due to irAEs developed hepatotoxicity, which was associated with shorter OS, contrasting with other irAEs. Because the occurrence of toxicity that requires nivolumab + ipilimumab termination is related to an increased risk of death, we may speculate that hepatotoxicity, in particular, impedes therapeutic benefits. Other real-world studies reported a 7–35% incidence of hepatotoxicity with the nivolumab + ipilimumab regimen, with G3-4 events in 4–24% of the cases [10, 14, 21, 22]. In a pivotal trial, all-grade ir-hepatitis were documented in 19% of patients, with G3-4 events reported in 8.6% of patients [29]. Our findings align with these reports, although further discussion is needed to understand the impact of hepatotoxicity on treatment outcomes.

Data from the US Food and Drug Administration’s AE Reporting System (FAERS) [30], which analysed 9,217,181 cases of adverse drug reactions, identified ICIs (particularly a combination of nivolumab+ipilimumab) as significant contributors to liver injury compared with other drugs. Patients with preexisting non-alcoholic fatty liver disease treated with single-agent ICIs are at increased risk of hepatotoxicity, possibly due to increased free radical and neoantigen production [31]. Similarly, chronic use of proton pump inhibitors is associated with a 13-fold greater risk of liver injury (HR 13.22, 95% CI 3.11–56.10), likely due to gut microbiota dysregulation [32]. We did not record these factors in our analysis.

Guidelines for the management of ir-hepatitis have been widely disseminated by international organizations, including ESMO [33], the American Society for Clinical Oncology [34], and the National Comprehensive Cancer Network [35], and are readily accessible in the literature. Therefore, they are not the focus of this paper. However, it is important to note that the majority of ir-hepatitis cases are asymptomatic, with clinical symptoms such as fever, malaise, abdominal pain, jaundice, and anorexia being relatively rare. Close monitoring of patients is essential to identify those who may require corticosteroid treatment or are resistant to steroids, in which case mycophenolate mofetil is recommended. Agents like infliximab, however, are not advised due to concerns regarding liver toxicity. For G1 ir-hepatitis, it is recommended to monitor liver enzymes every 1–2 weeks. In G2 cases, ICIs therapy should be temporarily withheld, and liver function should be monitored twice weekly. In G3-4 hepatitis, hospitalization and daily monitoring are necessary. If there is no improvement within 2–3 days of corticosteroid treatment, alternative immunosuppressive therapies such as mycophenolate mofetil or tacrolimus should be considered [33–35]. We hypothesize that the poor outcomes observed in our cohort with liver injury may be attributed to delayed corticosteroid administration or insufficient immunosuppression, as stronger immunosuppressants, such as mycophenolate mofetil, were only used in one case of hepatotoxicity.

In our study, patients who developed irAEs appeared to have better overall responses to therapy. However, given the retrospective nature of our data collection and the possibility of underreporting less severe irAEs, further prospective studies are needed to confirm this association. If irAEs can serve as predictive biomarkers for treatment efficacy, this could have significant clinical implications, potentially aiding in the early identification of patients who are more likely to benefit from ICIs. Nonetheless, more robust evidence is required before incorporating irAEs into routine clinical practice as prognostic or predictive markers.

### Study limitations

The main limitation of our study is the small sample size and the short follow-up period, which may affect the statistical power and generalizability of our findings. However, we aim to mitigate this limitation by continuing to monitor the enrolled patients over a longer period and by expanding the study to other centers across Poland. This approach will enhance the robustness of our conclusions and allow for a more comprehensive evaluation of the nivolumab + ipilimumab regimen in real-world settings. Yet, our study represents one of the largest real-world investigations of RCC patients treated with the nivolumab + ipilimumab regimen in a Caucasian population and is the first multicenter report of this kind in Poland. Additionally, the retrospective nature of data collection for some variables may have introduced biases, including potential inaccuracies in the recording of irAEs. Some irAEs, particularly those with nonspecific symptoms such as fatigue and musculoskeletal issues, may have been underreported due to their subtle presentation or overlap with other conditions. This underreporting could influence the reliability of our data on the incidence and severity of irAEs.

Furthermore, the findings should be interpreted with caution when considering their applicability to broader populations. Differences in genetic, environmental, and healthcare-related factors between populations may limit the direct generalizability of our results to non-Caucasian or non-Polish cohorts. Nonetheless, every report of post-registration clinical data, particularly in underrepresented populations, provides valuable insights to the existing literature.

## Conclusions

IrAEs may be considered a predictive factor for the nivolumab+ipilimumab regimen in RCC patients and typically occur during the first 2–3 months of treatment. Most toxicities during treatment with this regimen are mild or moderate. Hepatotoxicity, in particular, requires careful management, as it can significantly affect survival outcomes if not properly addressed.

Figure description.

## Electronic supplementary material

Below is the link to the electronic supplementary material.

Supplementary Material 1

## Data Availability

The datasets used and/or analysed during the current study are available from the corresponding author upon reasonable request.
