# Supplementary material for: Adverse events after nivolumab and ipilimumab combined immunotherapy in advanced renal cell carcinoma: a multicentre experience in Poland
Source: BMC Cancer. 2024 Nov 15;24:1411. doi: 10.1186/s12885-024-13192-8 (PMC11566080; doi:10.1186/s12885-024-13192-8)
Supplement: Supplementary file 1 — Supplementary Material 1 [file 12885_2024_13192_MOESM1_ESM.docx]

Supplementary materials

**Reimbursement criteria for nivolumab and ipilimumab combined immunotherapy in patients with renal cell carcinoma in Poland** [1].

1.1. General Eligibility Criteria

1. Histologically Confirmed Diagnosis:
   - Renal cell carcinoma with clear cell or sarcomatoid component for nivolumab with ipilimumab.
2. Advanced Tumor Stage: No possibility for radical local treatment.
3. No Prior Pharmacological Treatment: Advanced renal cancer untreated with systemic therapy, except prior cytokine-based immunotherapy for pazopanib.
4. Performance Status: Karnofsky score 70-100.
5. Age: 18 years or older.
6. Primary Tumor Removal: Previous tumor resection or documented multidisciplinary decision against nephrectomy.
7. Measurable Lesions: Objective assessment possible per RECIST criteria [2].
8. No Active Central Nervous System Metastases: Previous resection or stereotactic radiotherapy allowed if asymptomatic post-treatment.
9. Concurrent Active Malignancies: Treatment eligibility must consider prognosis related to coexisting malignancy.
10. No Significant Comorbidities: No clinical conditions contraindicating therapy per the current summary of product characteristics (SmPC).
11. Adequate Organ Function: Based on blood laboratory results per current SmPC.
12. Exclusion of Pregnancy and Breastfeeding: Confirmed non-pregnant and not breastfeeding.
13. Patient Agreement: Contraceptive use agreement per current SmPC of administered drugs.

1.2. Specific Eligibility Criteria for Therapy

1.2.4. Nivolumab with Ipilimumab

1. Intermediate or poor prognosis according to the IMDC scale.
2. No prior treatment with immune checkpoint inhibitors (anti-PD-1/PD-L1, anti-CTLA4), including adjuvant therapy.
3. No active autoimmune diseases, except Sjögren's syndrome, vitiligo, type I diabetes, hypothyroidism (treated only with hormone supplementation), psoriasis not requiring systemic treatment.
4. No systemic corticosteroids (exceeding 10 mg prednisone daily or equivalent) or immunosuppressive drugs within 14 days before starting treatment (inhaled corticosteroids are allowed).
5. No history of hypersensitivity to monoclonal antibodies.

2. Treatment Duration in the Program

Treatment continues until the attending physician decides to exclude the patient from the program, according to exclusion criteria.

3. Exclusion Criteria from the Program

1. Documented disease progression per current RECIST criteria [2] (for nivolumab with ipilimumab, confirmation of progression in another assessment after 4-8 weeks is possible).
2. In the case of oligoprogression (progression in no more than 5 foci in no more than 3 organs, including the CNS), treatment continuation is allowed if radical local treatment (surgery, stereotactic radiotherapy) is applied to these foci, provided the patient benefits clinically and other lesions remain responsive or stable.
3. Hypersensitivity to any active substance or excipient.
4. Pregnancy or breastfeeding.
5. Life-threatening toxicity or significant clinically relevant lower-grade toxicity that recurs despite appropriate management per current SmPC or oncology society guidelines.
6. Persistent significant deterioration in general performance or quality of life preventing treatment continuation.
7. Occurrence of diseases or conditions that, in the attending physician's opinion, preclude treatment.
8. Non-cooperation or non-compliance with medical recommendations, particularly regarding periodic control examinations to assess treatment efficacy and safety.

4. Continuation of Patient Treatment After Funding Ends in Non-standard Chemotherapy

1. As of April 1, 2018, patients whose treatment was funded under the non-standard chemotherapy program until that date may be qualified for the program.
2. The qualification mentioned in point 1 applies to treatment with the same active substance funded under the non-standard chemotherapy program.
3. Patients mentioned in point 1 do not need to meet other eligibility criteria at the time of qualification.
4. Patients mentioned in point 1 continue treatment in the program until the attending physician decides to end treatment with the given drug.

Additionally, patients treated with active substances funded in the drug program through other financing methods, except clinical trials for first-line treatment, are eligible for the drug program, provided they met the eligibility criteria at treatment initiation.

1. Dosage

The method of administration and any temporary treatment interruptions should follow the current Summary of Product Characteristics (SmPC) or accepted clinical practice. Dose reductions of the medications are permissible according to the current SmPC.

6. Qualification Tests for Treatment with Nivolumab in Combination with Ipilimumab

1. Histopathological diagnosis of renal cell carcinoma;
2. Complete blood count with differential;
3. Serum creatinine level;
4. Serum bilirubin level;
5. Alanine aminotransferase (ALT) activity;
6. Aspartate aminotransferase (AST) activity;
7. Lactate dehydrogenase (LDH) activity;
8. TSH and fT4 levels – for ipilimumab with nivolumab, cabozantinib;
9. Corrected serum calcium level;
10. Urinalysis – for cabozantinib;
11. Alkaline phosphatase activity;
12. Serum cholesterol and triglycerides levels – for temsirolimus;
13. Blood glucose level;
14. Other laboratory tests if clinically indicated;
15. Pregnancy test for women of childbearing potential (if clinically indicated);
16. Electrocardiogram (ECG);
17. Blood pressure measurement;
18. CT scan of the chest and abdomen;
19. Chest X-ray – only if it allows measurement of lesions and assessment of treatment response;
20. CT or MRI of the brain – for patients suspected of central nervous system metastases (i.e., with CNS symptoms or after prior local treatment of metastases);
21. Other imaging studies if clinically indicated.

Initial imaging studies must enable later objective assessment of treatment response according to current RECIST criteria.

7. Monitoring Treatment Safety

1. Complete blood count with differential;
2. Serum creatinine level;
3. Serum bilirubin level;
4. Alanine aminotransferase (ALT) activity;
5. Aspartate aminotransferase (AST) activity;
6. Urinalysis – for cabozantinib;
7. Serum cholesterol and triglycerides levels – for temsirolimus;
8. fT4 and TSH levels – for ipilimumab with nivolumab, cabozantinib;
9. Blood pressure measurement;
10. Other tests if clinically indicated.

Tests are conducted every 3-6 weeks – during combination therapy with ipilimumab and nivolumab.

Safety monitoring tests can be performed more frequently if clinically indicated.

8. Monitoring Treatment Effectiveness

1. CT or MRI of the relevant area;
2. Chest X-ray – if no CT scan is performed;
3. Other imaging studies if clinically indicated.

Tests are conducted:

1. At least every 12 weeks (with possible delay up to 2 weeks if treatment interruptions occur);
2. If treatment extends beyond 3 years, imaging tests can be performed at least every 6 months (with possible delay up to 4 weeks if treatment interruptions occur);
3. At the time of program discontinuation, unless due to documented disease progression, and always if clinically indicated.

The conducted imaging studies must enable objective assessment of treatment response. Treatment response evaluation should follow current RECIST criteria [2]. Monitoring the effectiveness and safety of treatment for patients mentioned in section Beneficiaries, item 4 should be conducted the same as for other patients.

9. Program Monitoring

1. Collecting treatment monitoring data in medical records and presenting them on request to National Health Fund (NHF) inspectors;
2. Updating data in the electronic drug program monitoring system available via an internet application provided by NHF, as per program description and at treatment termination;
3. Reporting and billing information to NHF (in paper or electronic form) as per NHF requirements;
4. For patients mentioned in section Beneficiaries, item 4, point 1, no data and information updates are required in the electronic drug program monitoring system.

**Definitions**

Efficacy parameters

- Overall survival (OS) - the duration from the initiation of combined immunotherapy to the patient's death;
- Progression-free survival (PFS)- the period from the onset of combined immunotherapy to PD on CT scan or patient death;
- Overall response rate (ORR), comprising complete remission (CR) or partial response (PR)
- Disease control rate (DCR), encompassing CR, PR, and stable disease (SD) (both according to RECIST 1.1 [2]

Comorbidities [3]

- Arterial hypertension- history of blood pressure > 140/90 mmHg or current antihypertensive treatment)
- Hypercholesterolemia - a total serum cholesterol > 5.2 mmol/L or ongoing antihypercholesterolemic therapy
- Renal insufficiency- glomerular filtration rate [GFR] lower than 90 but higher than 60 mL/min/1.73 m^2^
- Heart failure - ejection fraction < 50%
- Ischemic heart disease - symptoms of chest pain or myocardial infarction in the past
- Autoimmunologic diseases - recorded in patients’ history,
- Hypothyroidism - current use of hormone replacement therapy
- Type 2 diabetes mellitus - use of insulin or oral hypoglycemic agents, or fasting serum glucose > 7.0 mmol/L

**Table 1S.** **Additional information regarding baseline clinical characteristics of the enrolled patients.**

|  | All patients n=88 | irAEs  n=50 | no-irAEs  n=38 | p-value |
| --- | --- | --- | --- | --- |
| **Comorbidities** | | | | |
| Hypertension, n(%) | 47 (53.4) | 28 (56) | 19 (50) | 0.67 |
| Ischemic heart disease, n(%) | 10 (11.4) | 8 (16) | 2 (5.3) | 0.18 |
| Heart failure, n(%) | 5 (5.7) | 4 (8) | 1 (2.6) | 0.38 |
| Atrial fibrillation, n(%) | 5 (5.7) | 2 (4) | 3 (7.8) | 0.64 |
| Hypercholesterolemia, n(%) | 15 (17) | 11 (22) | 4 (10.4) | 0.25 |
| Hypothyroidism, n(%) | 18 (20.7) | 11 (22) | 7 (18.4) | 0.79 |
| Diabetes mellitus type 2, n(%) | 12 (13.6) | 9 (18) | 3 (7.9) | 0.22 |
| Venous thromboembolism, n(%) | 6 (6.8) | 6 (12) | 0 | 0.03* |
| Other malignancies, n(%) | 4 (4.5) | 1 (2) | 3 (7.9) | 0.31 |
| **Site of metastasis at the baseline computed tomography scan** | | | | |
| Nonregional Lymph nodes, n(%) | 35 (39.8) | 19 (38) | 16 (42.1) | 0.83 |
| Suprarenal glands, n(%) | 11 (12.5) | 7 (14) | 4 (10.5) | 0.75 |
| Liver, n(%) | 21 (23.9) | 12 (24) | 9 (23.7) | 1 |
| Central nervous system, n(%) | 6 (6.8) | 3 (6) | 3 (7.9) | 1 |
| Lungs, n(%) | 62 (70.5) | 37 (74) | 25 (65.8) | 0.48 |
| Bones, n(%) | 27 (30.7) | 15 (30) | 12 (31.6) | 1 |

Categorical variables are presented as numbers (percentages).
Abbreviations: n-number

**Table 2S. Univariate and multivariate analyses of factors potentially influencing treatment outcome.**

|  | Progression-free survival | Overall survival | |
| --- | --- | --- | --- |
|  | Univariate | Univariate | Multivariate |
| Age ≥ 65 | 0.5 (0.3-1), p=0.07 | 2.4 (0.9-6.8), p=0.09 |  |
| Performance status ≥2 vs. <2 | 1.1 (1.5-2.6), p=0.8 | 3.3 (1.3-8.4), p=0.013* | 3.8 (1.4-10.4), p=0.01* |
| KPS <80% vs. >80% | 1.3 (0.6-2.7), p=0.5 | 2.9 (1.2-7.2), p=0.22 |  |
| IMDC poor vs. intermediate | 1.9 (0.9-3.7), p=0.07 | 2.4 (0.9-5.9), p=0.06 |  |
| Time from the diagnosis to treatment onset <1 year vs. >1 year | 1.1 (0.4- 2.8), p=0.8 | 1.7 (0.4- 7.4), p=0.5 |  |
| Hemoglobin < unl vs. > unl | 1.1 (0.5-2), p=0.89 | 1.7 (0.6-4.4), p=0.3 |  |
| Neutrophils >unl vs. <unl | 0.7 (0.1- 4.9), p=0.7 | 1.4 (0.2-10.3), p=0.8 |  |
| Platelets >unl vs. <unl | 1.4 (0.7- 3), p=0.4 | 0.6 (0.2-), p=0.4 |  |
| Nephrectomy vs. no | 1.4 (0.5-3.6), p=0.49 | 0.8 (0.3-2.1), p=0.6 |  |
| Number of disease sites ≥ 2 vs. <2 | 1.6 (0.8-3.1), p=0.2 | 1.3 (0.5-.2), p=0.6 |  |
| Liver metastases vs. no | 0.9 (0.4-2.1), p=0.8 | 1.1 (0.3-3.3), p=0.9 |  |
| Central nervous system metastases vs. no | 1.4 (0.3-5.9), p=0.6 | 4.2 (1.2-14.6), p=0.03 * | 6.7 (1.8- 25.5), p=0.005* |
| Lungs metastases vs. no | 1.1 (0.5-2.1), p=1 | 1.7 (0.5- 5.8), p=0.4 |  |
| Bones metastases vs. no | 1.7 (0.9-3.5), p=0.1 | 1.3 (0.5-3.4), p=0.6 |  |
| irAEs vs. no-irAEs | 0.4 (0.2-0.9), p=0.018 * | 0.9 (0.4-2.3), p=0.9 |  |
| Endocrine irAEs vs. no | 0.5 (0.2-1.3), p=0.2 | 0.4 (0.1-1.6), p=0.2 |  |
| Hepatic irAEs vs. no | 0.7 (0.3- 1.9), p=0.5 | 2.6 (1.1-7), p=0.046* | 2.5 (0.9-6.9), p=0.07 |
| Cutaneous irAEs vs. no | 0.9 (0.3-.3), p=0.8 | 0.3 (0.03-1.9), p=0.2 |  |
| Number of irAEs ≥2 vs. <2 | 1 (0.4- 2), p=0.9 | 1.3 (0.5- 3.5), p=0.6 |  |
| Immunosupresive treatment vs. no | 0.4 (0.1-1.4), p=0.2 | 0.9 (0.3- 3.2), p=0.9 |  |
| Radiotherapy during immunotherapy vs. no | 1 (0.4-2.1), p=1 | 0.9 (0.3- 2.5), p=0.9 |  |

Abbreviations: irAEs- immune-related adverse events; n- number, IMDC- International Metastatic Renal Cell Carcinoma Database Consortium Risk Model, KPS- Karnofsky performance score, unl- upper normal limit

References

1. Obwieszczenie Ministra Zdrowia z dnia 18 marca 2024 r. w sprawie wykazu refundowanych leków, środków spożywczych specjalnego przeznaczenia żywieniowego oraz wyrobów medycznych na 1 kwietnia 2024 r. - Ministerstwo Zdrowia - Portal Gov.pl. https://www.gov.pl/web/zdrowie/obwieszczenie-ministra-zdrowia-z-dnia-18-marca-2024-r-w-sprawie-wykazu-refundowanych-lekow-srodkow-spozywczych-specjalnego-przeznaczenia-zywieniowego-oraz-wyrobow-medycznych. Accessed 19 May 2024.

2. Somarouthu B, Lee SI, Urban T, Sadow CA, Harris GJ, Kambadakone A. Immune-related tumour response assessment criteria: a comprehensive review. Br J Radiol. 2018;91.

3. Podręcznik Interna. https://www.mp.pl/interna/. Accessed 7 Aug 2024.
